# Observations on Excision of the Superior Maxillary Bone

**Published:** 1853-01

**Authors:** S. D. Gross

**Affiliations:** Professor of Surgery in the Medical Department of the University of Louisville.


### SELECTED ARTICLES.

### ARTICLE XIII.

*Observations on Excision of the Superior Maxillary Bone: Illustrated by seven Cases.* By S. D. GROSS, M. D., Professor of Surgery in the Medical Department of the University of Louisville.

(Continued from Journal, p. 151.)

### *Cases of Excision of the Upper Jaw.*

I shall close these remarks with a history of seven cases of excision of the upper jaw, which have been drawn up with great care, and the results of which have been satisfactorily ascertained in every instance.

CASE I.—The following case is remarkable, chiefly on account of the cause of the tumor, which was quite small. Miss C., aged twenty-one, of Frankfort, Kentucky, came under my observation in June, 1843, for an enlargement of the left upper jaw-bone, which she first noticed about two years and a half previously; it occupied the alveolar process, was free from pain and discoloration, and produced no appreciable prominence on the corresponding side of the face until eighteen months ago. About six months after the tumor first attracted attention, the first bicuspid tooth became loose, and was extracted by a dentist; the operation was followed by considerable hemorrhage, but this soon ceased, and the parts healed in a short time. After this, the tumor increased a little more rapidly, and by degrees the second bicuspid became loose; but, as it did not ache, it was not removed.

At the time of Miss C's visit, the tumor was about the volume of a large hickory nut, and seemed to be formed at the expense mainly of the outer surface of the alveolar process; it was hard and inelastic, devoid of pain or soreness, and unaccompanied by any disease of the gum. The general health was, and, in fact, always had been, excellent.

As the tumor was making steady, though slow, progress, and was productive of some deformity of the lip, as well as of great mental disquietude, I determined at once to remove it. As a preliminary measure, the canine tooth was extracted by my friend, Dr. Somerby. The bone was then sawed through, at the site of this tooth, as far as the inner table of the alveolar process, and to the same extent at the posterior limit of the tumor, which was then razed with a chisel and a strong knife, the lip having been previously detached from its upper extremity. As the internal lamella of the bone was perfectly sound, it was, of course, permitted to remain, especially when it was ascertained that the whole trouble had been occasioned by the presence of a tooth, apparently a cuspid, a little smaller than natural, but well formed, with the crown reversed, or directed upwards towards the antrum of Highmore. The parts soon healed after the operation, and the patient recovered with hardly any defect, except what resulted from the extraction of the tooth at the time of the operation. Could the real nature of the tumor have been determined before the operation, this tooth would, of course, have been saved.

Supernumerary teeth are not of unfrequent occurrence, especially in the upper jaw; they are much more common among the permanent than the temporary set; and their shape generally resembles the cuspid or canine teeth. They are not, however, confined to the front of the jaw, though they are more frequent here than elsewhere. Inverted teeth are seldom met with. Dr. Somerby, and Dr. Goddard, two eminent and experienced dentists of this city, inform me that they have never met with an example. Mr. Hunter,* gives a drawing of the upper jaw of a child, in which the cuspid tooth was inverted, "so that its point was turned up against the jaw, and the growing mouth of its cavity towards the gum." Albinus relates a singular instance where a tooth was found on each side in the ascending process of the maxillary bone, between the nose and the orbit; it was long and very thick, and situated in such a manner that the crown looked upwards towards the eye. No satisfactory explanation has yet been furnished of the manner in which this vicious direction of the teeth is brought about. The subject is interesting in connection with that of osseous tumors of the superior maxillary bone.

Case 2.—Miss B., aged nineteen, of a florid complexion, and rather delicate health, from the vicinity of Shelbyville, Kentucky, observed, nine months ago, several weeks after a severe attack of bilious fever, a small tumor in the right upper jaw, which gradually increased in size, and at length formed a slight prominence under the cheek. At her visit to me in April, 1845, the parts exhibited the following appearances: the tumor, which was firm and solid, projected internally a little beyond the middle line, while at the side it extended from the anterior edge of the lateral incisor, to the horizontal plate of the palate bone. The alveolar process was much expanded, and the middle grinder, having become loose, had been extracted by the family physician, under the belief that the disease was an abscess of the antrum of Highmore. The other teeth were sound, and firm in their sockets. The integuments of the cheek and the mucous membrane of the mouth were healthy. The speech and deglutition seemed to be little, if at all, affected. The tumor was the seat of slight darting pains, which had latterly become more constant and severe, and its volume had nearly doubled itself during the last eight or nine weeks. The general health had not materially suffered.

Having properly prepared the system, I performed the ope-

*Natural History of the Teeth, tab. 8, fig. 9.

ration of excision of the affected parts on the 19th of April, 1845, in the presence of Drs. Johnston, Cochran, Somerby, Colescott, and T. L. Caldwell, the latter three of whom afforded me their kind assistance. The patient being a woman of unusual fortitude and determination, sat upon a chair, with her head reclining against the breast of one of the gentlemen just named. As a preliminary step, Dr. Somerby, extracted the lateral incisor and last molar tooth. An incision was then made over the prominent portion of the swelling, beginning at the commissure of the mouth, and terminating near the outer angle of the eye, its direction being slightly curvilinear. The two flaps being next detached, and held out of the way, the jaw was divided, with a Heys' saw, by the side of the middle incisor, from whence the instrument was carried through the horizontal plate, close along the raphe, as far back as the palate bone. The tumor was then attacked with the chisel and mallet by cutting through the outer wall of the antrum, the cavity of which was occupied by a soft fungous mass, and was evidently the original seat of the whole mischief. This being thoroughly cleared out with the lenticular and handle of the scalpel, the operation was completed by sawing the jaw at its junction with the malar and palate bones. An artery, a branch of the one which supplies the vomer, was cut, and required torsion. No vessels were tied. The quantity of blood lost did not exceed four or five ounces. The woman bore the operation admirably.

The cavity left by the removal of the tumor, was filled with patent lint, and the wound closed with four twisted sutures, aided by a compress and roller. As there was a good deal of pain, a grain of morphia was administered after the girl went to bed.

Everything went on well until the fourth day, when erysipelas, which had been for some time epidemic in the city, made its appearance at the upper angle of the wound, and gradually extended over the whole face, ears, and forehead. Under the influence of a few active mercurial cathartics and the application of the tincture of iodine, the disease was soon arrested, and comfort restored to the part and system. The sutures were removed on the fourth day, when the union was found to be complete.

As soon as suppuration was fully established in the bony cavity of the mouth, the lint was removed, and after the parts were well syringed with a solution of chloride of soda, it was replaced, to afford support to the cheek. These dressings were renewed daily for upwards of a week.

Miss B. left Louisville in a fortnight after the operation, almost as well as ever, and with hardly any perceptible scar, so perfect was the union of the wound. There was, of course, a considerable retrocession of the cheek, and great impediment in articulation; the bottom of the osseous cavity was covered with healthy granulations, and there was still some discharge of pus.

The tumor was carefully examined after removal, and exhibited all the characteristic features of encephaloid. Indeed, no morbid growth that I have ever inspected was better marked in this respect.

I saw this young lady four years after the operation, and am happy to say that she is still living, her residence being the neighborhood of Paducah. Her articulation is greatly improved, and the deformity of the face is so slight as to escape casual observation.

Case 3.—Mrs. Susan Green, aged thirty-eight, a married woman, the mother of nine children, from Crawford county, Indiana, visited Louisville in June, 1847, to consult me respecting a tumor of the left superior maxillary bone, which had of late become a source of much distress to her. Her statement was that the affection was first noticed in 1830, as a small, movable lump, situated opposite the second large grinder, between the median line and the alveolar process. The growth was slow and gradual until about four years ago, when, after cauterizing it, for the third time, with nitrate of silver, it began to advance very rapidly, and has continued so until the present time. The tumor is now as big as an ordinary fist, and produces great distortion of the features: protruding the ball of the eye, pushing out the cheek, and closing the left nasal cavity. Internally it overlaps the right raphe of the roof of the mouth, while posteriorly it projects slightly beyond the maxillo-palatine suture, displacing the tongue, and thrusting it over towards the right side. Mastication and deglutition are both considerably impeded. The tumor is hard, firm, and inelastic. Last winter, the two bicuspid and two anterior molar teeth became loose, and were extracted, without causing any particular pain, or serious hemorrhage.

Until about six months ago, the tumor was free from suffering, except that it was the seat, pretty constantly, of a feeling of tension or tightness. Since then it has become affected with a dull aching pain, which radiates about in different directions, and is particularly severe in the left malar bone, and corresponding nostril. Mrs. Green is not aware that her jaw has ever received any injury, and she knows of no cause of the disease. Until a few months ago, her health was always remarkably good; of late, she has lost flesh, and her countenance has assumed a rather sallow aspect.

Excision of the tumor was performed on the 15th of June, with the aid of my pupils, Dr. Summers, Dr. Bozeman, and Dr. Ector, and in the presence of a number of physicians and students. Dr. Goddard had the kindness to extract, as a preliminary measure, the canine and last molar teeth. The patient being placed upon a table, with her head resting upon a pillow, and being fully under the influence of ether, an incision was made over the prominent portion of the tumor, from the left angle of the mouth to within a short distance of the outer canthus of the eye, in a slightly curvilinear direction, the convexity of the curve looking downwards. The flaps being dissected off, the saw was applied to the anterior and lateral part of the jaw, from whence it was carried obliquely backwards through the raphe of the horizontal lamella of the maxillary bones to a little beyond their junction with the palate bones. Continuing the operation, I next divided, with the saw and pliers, the root of the ascending process of the bone, nearly on a level with the floor of the orbit, and finally the malar process and a part of the horizontal plate of the palate bone. Having thus severed its principal and most important connections, the tumor was wrenched from its bed with the hand and chisel, and the bottom and sides of the chasm immediately cleared of everything betraying the slightest appearance of disease. A good deal of oozing of blood. took place at this stage of the procedure, but this soon ceased spontaneously, and did not afterwards recur.

The osseous cavity being stuffed with lint, wet with a saturated solution of alum, the edges of the wound were brought together by five twisted sutures, and the woman, somewhat exhausted by the operation, removed to her bed. Not more than five or six ounces of blood were lost during the operation, which occupied but a few minutes. No arteries, except a small muscular branch, required the ligature. Some vomiting occurred at the close of the operation, from the effects, apparently, of the ether. As soon as this had passed off, a grain of morphia was given, to allay pain, which was now considerable, and mustard was applied to the chest and extremities.

It is not necessary to present a diary of the symptoms and treatment of this case, which progressed most favorably, with the exception of a slight attack of erysipelas of the face, from the time of the operation till the patient left Kentucky, nearly three weeks after. The sutures were withdrawn at the end of the fourth day, when the wound was found to have united, in its whole extent, by the first intention. The lint was removed from the mouth as soon as the suppurative process had fairly begun, and was reinserted daily for a week, when its employment appearing no longer necessary, it was dispensed with. As a detergent, free use was made of the chloride of lime in solution. When the patient went home, the bottom of the chasm was covered with fine healthy granulations, and nearly all the discharge of pus had ceased. The face was but slightly disfigured. The voice was, of course, materially changed, and there were many words which she was unable to pronounce with distinctness.

Having learned that Mrs. Green had suffered a relapse, and that she had become a patient of Dr. Sloan, of New Albany, I addressed a note to that gentleman, who kindly furnished me with the following facts: "Mrs. Green called upon me on the 4th of March, 1849, with a considerable enlargement of the median portion of the alveolar process of the left upper jaw, which I removed the next day, by making a vertical incision from below the inner angle of the eye to the border of the left lip, detaching the ala and septum of the nose, and then cutting the bone obliquely across from between the incisors on the right side, through the nasal cavity, to the opening made in your operation. The piece thus removed contained, I think, the roots of the canine and two left incisor teeth, with the central incisor of the right side.* About eight months after, she applied to me for a small growth on the posterior boundary of the cavity, which I removed with its bony attachments.

I saw Mrs. Green again in May, 1852. Her disease had again returned, involving the posterior wall of the antrum, the palatine arch, and the pterygoid process, and extending towards the coronoid process of the lower jaw. I expect she will visit me next autumn."

Case 4.—William Collinridge, aged sixty-six, a brass-founder by occupation, and a married man, of temperate habits, and nervo-sanguineous temperament, was recommended to me by his professional adviser, Dr. Hagan, of this city, in the latter part of July, 1847, on account of a tumor in the right upper-jaw. He had always been healthy, until about four months ago, when he became feeble and nervous, and soon after discovered a slight swelling of the gum, attended with aching of the large grinders. His suffering increasing, he consulted a physician, who advised the extraction of the affected teeth, which was accordingly done, but with only temporary relief. The operation was attended with considerable hemorrhage, and was soon followed by an increase of the swelling, and a recurrence of the pain, in all its previous violence.

* Dr. Sloan is, I think, in error in saying that the piece included the canine teeth, as I have a distinct recollection that I sawed the bone through the socket of that tooth, in my operation.

When Collinridge first consulted me, the tumor was so large as to form quite a prominence under the cheek, and to encroach a good deal upon the buccal cavity; his speech was thick and indistinct, and he was unable to masticate solid food. The enlargement extended from the lateral incisor to the posterior extremity of the bone, and had a softish and elastic feel, not unlike an encephaloid tumor. The mucous investment was remarkably florid and congested, but sound in texture, and the integuments of the face were perfectly healthy. The pains were excessive, and almost without intermission; darting about in different directions, and usually most severe at night and during damp and cloudy states of the weather. All the teeth on the right side, excepting the two incisors, had either dropped out or been extracted.

On the 9th of August, 1847, assisted by Dr. Hagan and several of my private pupils, I excised the diseased mass, the patient having previously inhaled ether. The incision was made, as in the cases already described, from the commissure of the lips in a slightly curved direction, to within a short distance of the external angle of the eye. The flaps being dissected up, and held out of the way, the saw was applied in front, close by the central incisor, and thence carried back to the palate bone. The bone was then cut at its junction with the malar bone, at the ascending process, and along the orbitar plate, when the whole mass was forced out of its bed. A considerable flow of blood took place, at this stage of the operation, which had to be stopped for a few minutes, until the patient recovered from the effects of the resulting exhaustion. A large sponge was immediately pressed upon the bleeding surface; and as soon as I was able to proceed, the blood was wiped away, and the osseous cavity cleared of its morbid contents with the knife, chisel, and lenticular. The hole was then stuffed with lint, wet with a saturated solution of alum, and the edges of the wound were approximated by a compress and bandage. Only two small arterial branches required the ligature.

Nothing of any special interest occurred after the operation, which was not followed by a single bad symptom. The wound united by the first intention, and the sutures were removed at the end of the fifth day. Gradually granulations sprung up at the bottom of the gap, greatly diminishing its depth and extent, and thus aiding in the recovery of the power of speech and deglutition. The general health rapidly improved, and the patient was perfectly free from the local suffering which had so much harrassed and distressed him before the operation. Things went on in this manner for about six weeks, when, all at once, a small tubercle made its appearance upon the skin, at the former site of the tumor, and soon acquired the size of a twenty-five cent piece; it was hard and slightly elastic to the touch, projected some distance above the surrounding surface, was of a dark livid hue, and the seat of a constant sharp, shooting pain. No attempt was made to remove this, for it was but too evident that it was of a malignant character, and that any further operation would be worse than useless, the more especially as the general health now began to give way, and the granulations at the bottom of the wound presented a pale, flabby, and unnatural aspect. Soon after this, small tubercles, of a kind similar to that just mentioned, showed themselves upon the temple, rapidly increasing in size, and greatly aggravating the local distress. The pains were excessive, shooting and darting about in every direction, through the face and head, and requiring the constant use of morphia for their subjugation. The tumor on the cheek acquired a large bulk, and at length ulcerated, discharging a large quantity of sanguinolant fluid, and occasionally pure blood. Excessive emaciation supervened, followed by hectic irritation, and colliquative diarrhea, under which the patient sunk in October, 1848, fourteen months after the operation. No examination was made.

The morbid mass, in this case, possessed all the external features of encephaloid. The bone was deprived of its normal texture, and the soft portion of the tumor was of the color and consistence of the adult brain.

Case 5.—The subject of this case was Sylvia, a negress, married, thirty-five years of age, and a servant of Mr. Stephen Brown, of Springfield, Kentucky. She came under my care in 1849.

The disease first appeared about three years ago, as a boil, on the inside of the gum, and has gradually but steadily attained its present dimensions. During the last few months, however, its growth has been much more rapid than before, and this, so far as can be ascertained, without any assignable cause. The the tumor, at this time, embraces the jaw, from the right canine tooth to the malar articulation, filling up the zygomatic fossa, and hideously distorting the features. In front, it does not quite reach the middle line, but posteriorly it projects beyond the raphe, and extends as far back as the soft palate, which, together with the side of the pharynx, is red and tumid, causing much difficulty of articulation and deglutition. The largest portion of the tumor, however, is external to the jaw, within the lips and cheek, reaching from the zygomatic process of the temporal bone to the angle of the inferior maxillary bone behind, and to the anterior portion of the mouth in front. In its shape it is irregularly globular, its longest diameter, the antero-posterior, being about three inches and a half. Of a pearly pink appearance on the inside, it is of a deep red, almost purple color, externally beneath the cheek, and is traversed by numerous vessels, filled with dark blood. In its consistence it is hard and inelastic, except along the groove formed by the pressure of the teeth of the lower jaw, where it is somewhat soft, and ulcerated. It has been more or less painful ever since its commencement, especially at the back part. Much distress has also been experienced, of late, in the forehead, temples, and nape of the neck.

During the progress of the tumor, the back molar tooth became loose, and was extracted, showing a small abscess filled with purulent matter. The operation afforded only temporary and partial relief, and was attended with a good deal of hemorrhage. Subsequently, the other grinders shared the same fate. A neighboring physician, supposing that the main disease was in the antrum, punctured it with a lancet, but nothing but blood issued from the wound.

About three months before her arrival in Louisville, a hard, circumscribed swelling, as I am informed by Dr. Hughes, of Springfield, appeared on the front of the neck, just above the sternum, slightly compressing the trachea, and impeding respiration. After sometime, fluctuation was perceived, when it was opened, and soon after it gradually went away, except a little remnant, which is still discharging a thin, sanious fluid.

Although my patient's health was a good deal impaired, and the tumor was of an unpromising character, I considered it my duty to operate, being satisfied that, bad as the chances were for recovery, the case was not wholly hopeless. Accordingly, on the 12th of April, assisted by Drs. Colescott, T. G. Richardson, Henry and Thomson, the woman was placed upon the table, and put under the influence of chloroform, the external lateral incisor having been previously extracted by Dr. Goddard. The external incision, curvilinear in its direction, extended from the commissure of the lips to within a few lines of the outer angle of the eye, over the most prominent portion of the morbid mass, the surface of which was fully exposed by the reflection of the two flaps of integuments. The bone being divided in front and internally, with a pair of sharp pliers, and the anterior wall of the antrum laid open with a stout knife, the tumor was turned out of its bed, followed instantly by a copious gush of blood, and the fainting of the patient. Pulling the pillow away from her head, and thrusting a sponge into the bottom of the chasm, I scraped out, with all possible haste, the remnant of soft structure, and then sawed the malar and palate bones; finishing the operation by dissecting out a process of the tumor which had dipped in between the arch of the palate and the pterygoid muscles, between the upper and lower jaw.

Two small arteries being secured, the blood was sponged away, and the osseous cavity stuffed with lint, wet with a saturated solution of alum. The edges of the wound were approximated by four twisted sutures and collodion plasters, and the patient, much revived by means of hartshorn and brandy, put to bed. A grain of morphia was administered soon after the operation, and until the following morning the woman was most assidiously watched by my assistants, Drs. Thomson and Henry, whose kindness I had frequently witnessed on similar occasions. Complete reaction had taken place by 4 o'clock in the afternoon, four hours after the operation, and the night was passed in a comparatively tranquil and comfortable manner, there being but little pain and difficulty of deglutition. The pulse was eighty and soft; there was little thirst, and the woman had taken some gruel. The face and eyelids were slightly swollen.

On the 14th, two days after the operation, the patient had several free motions from the effect of an ounce of oil taken the previous evening; she had slept some, and there was no fever or thirst. The swelling of the face and eyelids was the same as on the 13th. On the 15th, the patient expressed herself quite comfortable, and the parts looked well. On the 16th, I withdrew the needles, and removed the lint from the mouth, The next day, the fifth from the operation, the collodion plasters came off, thus exposing the wound, which was found completely united by the first intention.

After this period no diary was kept; the case went on well; and, in less than three weeks, the woman returned to her master, rapidly improving in strength and flesh. Sometime afterwards, as I learn from Dr. Montgomery, of Springfield, the disease reappeared in the roof of the mouth, in the form of a small excrescence, which rapidly increased until it attained the volume of a pullet's egg. It was attended with severe pain in the head, and great swelling of the nose, which looked as if it were distended with a large polypus. The corresponding eye participated in the disease, and finally burst. There was great difficulty of breathing; and, about four weeks before the woman died, the disease showed itself in the forehead and opposite eye. Death took place on the 15th of December, 1850, twenty months after the operation.

CASE 6.—Benjamin Hudson, of Alabama, aged fifty-two, a farmer, married, and the father of eleven children, of a sanguineo-bilious temperament, dark complexion, and temperate habits, was always healthy until the latter part of November, 1850, when he perceived, without any assignable cause, a small circumscribed swelling in the right cheek, a short distance below the outer corner of the eye, and just within the malar bone.

The face was sore and tender to the touch, but the skin was free from discoloration. About three weeks after he observed this swelling, he noticed, for the first time, a little tumor, about the size of the end of a finger, hard and firm in its consistence, situated under the cheek in contact with the gum. In fact, it looked and felt like a gum-boil. It bled upon the slightest touch ; indeed, sometimes spontaneously; now slightly, and then quite profusely. Increasing gradually, but steadily, in volume, it at length encroached upon, and loosened, the anterior and posterior molar teeth, so as to render it necessary to extract them in February, 1851; the middle one having been removed about eighteen years previously, along with two small fragments of the jaw-bone. By the middle of April, the tumor had attained the volume of a goose's egg, and was making most rapid progress. The pain which attended it, was of a sharp, lancinating character, and was so violent and incessant as to deprive the patient almost entirely of appetite and sleep. He had, also, by this time lost a great deal of flesh and strength.

The patient reached Louisville on the 3d of May, 1851, in the following condition. The right cheek was occupied by an enormous tumor, of an elongated ovoidal shape, being longer in the antero-posterior than in the vertical diameter; it was very tender on pressure, and the seat of a severe and constant pain, sharp and darting in its character. The integuments were of a purple color, and the corresponding portion of the upper lip was very much swollen and distorted. The tumor extended up to the eye, backwards over the zygomatic process, forwards to within a few lines of the nose, and downwards towards the base of the lower jaw. Viewed within the mouth, it was found to reach inwards to the middle line, forwards to the lateral incisor, backwards to the soft palate, and downwards into the floor of the buccal cavity. It was of a pale reddish complexion, firm and elastic, tender on presure, and irregular in its shape. Towards its anterior and lateral aspects, where it lay in contact with the cheek, was an ulcer about the size of an American eagle, the surface of which was of a brownish color, rough, and fungous. It was free from pain and hemorrhage, and the patient was unconscious of its existence. The tumor seriously impeded articulation and deglutition, but the respiration was natural. The patient had no appetite, and he had not slept well since he left home; he was thin, and his countenance, although not sallow, had a haggard, care-worn expression.

Having purged and dieted my patient for nearly a week, I operated upon him in the presence of Drs. Hardin, Raphael, Thomson, Richardson, and other medical gentlemen. Chloroform was given him to the requisite extent; and, as a preliminary step, Dr. Goddard, extracted the central incisor. By means of a curvilinear incision, extending from the commissure of the lips to the zygomatic process, an inch and a half from the outer canthus of the eye, two flaps were marked out, which were then dissected up, and carefully held out of the way. The jaw being sawed through in front at the middle line, the instrument was then carried through the horizontal plate of the *left* jaw-bone, close along the raphe, and back through the entire length of the corresponding portion of the palate bone. The next step of the operation consisted in dividing the maxillary process of the malar bone, the periosteum of which appeared to be involved in the disease. The nasal process of the jaw bone being now sawed just below the lachrymal sac, the morbid mass was attacked with the chisel and mallet, and severed along with the orbitar plate, and the horizontal portion of the palate bone. The inferior turbinated bone, and a portion of the nasal septum were also detached. Finally, the operation, one of the most horrid I have ever executed, was completed by freeing the surface of the osseous cavity, with its various chasms and ramifications, of all diseased matter, with the knife, scoop, and lenticular.

The patient, towards the close of the operation, became faint, and was obliged to use cordials, and other restoratives. The loss of blood did not exceed eight or ten ounces; only a few little arteries required to be secured. The bony cavity was stuffed with lint, wet with alum water, and the parts were approximated with six twisted sutures and collodion strips, supported by a compress and bandage.

As soon as the effects of the chloroform had passed off, the patient took a grain of morphia. In the evening I learned that he had been quite comfortable nearly all the afternoon; he had slept some, and had little or no thirst, nor much local uneasiness. The following night was passed in tolerable comfort, next day he had some fever, and he complained of a sense of tension in the deep portion of the wound. On the third day, slight erysipelas came on in the right side of the face, and rapidly increasing in extent, soon spread over the forehead and temple. Recovering from this disease in a short time, every thing went on well for a fortnight, when the patient was seized with pneumonia, which gradually progressed from bad to worse, despite the means employed for arresting it, until the 28th of May, when it proved fatal. It is proper to observe, that the wound healed nearly in its entire extent by the first intention.

An examination, after death, revealed high inflammation of the lungs and pleura on both sides, with considerable effusion into the thoracic cavity. No carcinomatous disease was found any where, and the parts concerned in the operation, exhibited a perfectly healthy aspect.

Case 7.—In the following case, which came under my observation in May, 1851, only a portion of the upper jaw was removed, the disease necessitating the operation, having apparently commenced in the maxillary sinus.

J. H. P., of Henderson county, Kentucky, aged fifty-eight, a tall, slender, hard-working farmer, had his attention arrested in the early part of February, 1851, by a watering of the left eye, followed, soon after, by a severe, dull aching pain, confined, at first, entirely to that organ, but subsequently diffused over the upper part of the face, the forehead and temple. He had always enjoyed good health up to this time, and was not aware that he had received any local injury. About the middle of April, he first noticed a swelling in the lower part of the orbit, and also a free discharge of muco-purulent matter from the left nostril.

Mr. P. arrived in Louisville, accompanied by his physician, Dr. Howard, of Owensboro, on the 24th of May. The upper portion of the left side of the face was, at this period, affected with a tumor, which encroached so much upon the corresponding eye as to protrude it partially from its socket, and which was the seat of a sharp, lancinating pain, darting about in different directions, and so constant and intense as to deprive him, in a great measure, of appetite and sleep. The eye had a watery, inflamed appearance. The integuments of the face were in a sound state. Upon inspecting the left nostril, I found it occupied by a large swelling, evidently a portion of the tumor already described, and discharging a large quantity of thin bloody fluid, of the most fetid character. The general health was somewhat impaired, but the patient was in good spirits, and anxious for an operation.

Although I must confess that I did not like some of the symptoms of this case, and so expressed myself to Dr. Howard, yet such was the desire of the patient to be relieved, that I was at length prevailed upon to undertake an operation. Accordingly having dieted him for a few days, and given him some purgative medicines, I proceeded to my task on Tuesday, the 27th of May, aided by my friends, Dr. T. G. Richardson, Dr. Thomson, Dr. Hardin and Dr. Raphael. Chloroform having been administered, two incisions were made at right angles with each other, in the form of an inverted L, the limb of one extending along the side of the nose as low down as the lip, and that of the other along the margin of the orbit of the eye. The flap was then dissected up, and the wall of the antrum attacked with the saw, chisel, and lenticular, until the whole of the diseased structures, osseous and soft, was turned out. A process of the tumor dipped back deep into the orbit, and was removed with much difficulty, owing to the importance of the surrounding parts, and the manner in which they were obscured by the bleeding. The whole of the upper jaw, excepting the alveolar and horizontal portions, was cut away along with a part of the malar bone. A large artery, a branch of the internal maxillary, bled profusely at the bottom of the osseous cavity, and was finally, after much ado, secured with a ligature. In consequence of the great oozing of blood, it was necessary, before replacing the integuments, to stuff the cavity with lint, wet with a strong solution of alum. The edges of the flap were approximated with three interrupted sutures and collodion strips, supported by a compress and bandage.

The patient was put in bed, his head reclining upon a high pillow, and immediately took a grain of morphia. A short time after he lay down, blood was seen escaping from the nostril; but the quantity was small, and the discharge soon stopped of its own accord, and did not subsequently recur. At the end of the fifth day, free suppuration being established, the lint was carefully withdrawn through the nose, and through a small opening in the upper limb of the wound, the greater portion of which had united by the first intention. The cavity was afterwards syringed daily with a weak solution of chloride of soda, under the use of which, together with mild tonics and a nourishing diet, the parts rapidly healed, and the general health greatly improved. The patient left Louisville on the 8th of June, with directions to pay the utmost attention to himself in every particular. Within a few days after he reached home, he was seized with a violent attack of dysentery, which was then epidemic in that region of country, and under which he sunk, completely exhausted, on the 2d of July. Dr. Howard, of Owensboro, from whom I have learned these particulars, in a letter dated August 17, 1852, informs me that a tumor, about the volume of a medium-sized marble, and the seat of the most excruciating pain, appeared, about a week before he died, in the left orbit, just above the inner canthus.

I was not aware, until I received the above intelligence, that Mr. P. had died so soon after he left Louisville, and from a cause so entirely foreign to that of the disease for which I operated upon him. How long he might have lived, had he not been thus assailed, after the affection of the orbit showed itself, is a matter which must be left wholly to conjecture. Deducting this case from those which are stated, under the head of *results*, as having proved fatal, it will be perceived that only two died from the *immediate* effects of a relapse of the malady for which recourse was had to the knife.

It is proper to add that the tumor, in this case, exhibited all the physical and microscopical characters of encephaloid, as I was assured by my friend, Dr. T. G. Richardson, who carefully examined it within a few hours after its removal.

I had forgotten, until after the greater portion of this paper was printed, that Dr. Stevens, to whom, in common with some of my brethren, I have ascribed the credit of being the first to excise the upper jaw in this country, had been anticipated in this undertaking by Dr. Jameson, of Baltimore. In looking, accidentally, over the fourth volume of the American Medical Recorder, I find that this distinguished surgeon performed the operation in question in November, 1820, nearly three years prior to that of Dr. Stevens. The primitive carotid was tied as a precautionary measure, and the patient remained well when the case was reported, nearly three months after the operation. The account of the case is accompanied by a graphic engraving, showing the size and situation of the tumor, as well as the great deformity to which it gave rise.

*Louisville, Aug.* 24, 1852.

